# In Vitro Virucidal Effect of Intranasally Delivered Chlorpheniramine Maleate Compound Against Severe Acute Respiratory Syndrome Coronavirus 2

**DOI:** 10.7759/cureus.10501

**Published:** 2020-09-17

**Authors:** Jonna B Westover, Gustavo Ferrer, Hector Vazquez, Arian Bethencourt-Mirabal, Camille Celeste Go

**Affiliations:** 1 Animal, Dairy and Veterinary Sciences, Utah State University, Utah, USA; 2 Pulmonary and Critical Care Medicine, Pulmonary Institute, Aventura, USA; 3 Pulmonary and Critical Care Medicine, Dr. Kiran C. Patel College of Osteopathic Medicine, Nova Southeastern University, Fort Lauderdale, USA; 4 Pulmonary Critical Care, Aventura Hospital and Medical Center, Miami, USA; 5 Family Medicine, Larkin Hospital Palm Springs Campus, Miami, USA

**Keywords:** sars-cov-2, covid-19, nasal spray, chlorpheniramine maleate, virucidal

## Abstract

Background

The initial global outbreak of the novel coronavirus disease 2019 (COVID-2019) pandemic, which is responsible for the severe acute respiratory syndrome 2 (SARS-CoV-2), shares similarities with the severe acute respiratory syndrome coronavirus (SARS-CoV) and Middle East respiratory syndrome coronavirus (MERS-CoV) and behaves similarly to influenza with a high intranasal viral load. The genome sequence of COVID-19 opened the opportunity for multiple in vitro and clinical trials, but we still do not have a clear path to treatment. Chlorpheniramine maleate (CPM) is a safe and effective antihistamine with potent antiviral activity against various strains of influenza A/B, thus suggesting that CPM has broad antiviral activity. We tested the virucidal potential of CPM in a nasal spray composition currently in development as an anti-allergy medication.

Methods

The virucidal activity of CPM was tested using viral stock of SARS-CoV-2, USA-WA1/2020 strain in Vero 76 infected cells. The endpoint titer 50% cell culture infection dose (CCID_50_) values were calculated using the Reed-Muench (1948) equation. Three independent replicates of each sample were tested, and the average and standard deviation were calculated. Results were compared with untreated controls using one-way ANOVA (analysis of variance) with Dunnett’s multiple comparison test in GraphPad Prism (version 8) software.

Results

After 25 minutes of contact time, the nasal spray reduced the levels of the virus from 4.2 to 1.7 log10 CCID_50_ per 0.1 mL, a statistically significant 2.5 log reduction value or 99.7% reduction in the viral load.

Conclusions

This study demonstrates the strong virucidal effect against SARS-CoV-2 of a nasal spray containing CPM. Given that CPM has broad antiviral effects against influenza and virucidal effect against SARS-CoV-2, we propose two further studies: a randomized placebo-controlled study of intranasally delivered chlorpheniramine in patients with mild-to-moderate SARS-CoV-2 and a second study aiming to determine the potential antiviral and adjuvant effects of CPM plus hydroxychloroquine, versus hydroxychloroquine alone, in hospitalized patients with SARS-CoV-2.

## Introduction

The initial global outbreak of the novel coronavirus disease 2019 (COVID-2019) pandemic, which is responsible for the severe acute respiratory syndrome 2 (SARS-CoV-2), was first reported in Wuhan, China, at the end of December 2019 [1]. As of July 14, 2020, there were more than 13.3 million confirmed cases worldwide, with total deaths exceeding 573,000. SARS-CoV-2 shares similarities with severe acute respiratory syndrome coronavirus (SARS-CoV) and Middle East respiratory syndrome coronavirus (MERS-CoV) [2]. The genomic sequence of SARS-CoV-2 has enabled multiple in vitro and clinical trials aiming to help infected patients. Unfortunately, evidence supporting any of the proposed prophylactic or treatments is negligible.

In an attempt to repurpose existing drugs with antiviral potential that might be used in COVID-19 prevention and treatment, we tested the virucidal potential of chlorpheniramine maleate (CPM) in a nasal spray composition currently in development as an anti-allergy medication. CPM is a first-generation antihistamine that has been marketed in the United States for several decades and is currently widely used. Recently published literature suggests that CPM has strong antiviral and anti-inflammatory activity.

Chlorpheniramine is a safe and effective antihistamine whose main side effect is drowsiness. However, evidence suggests that intranasal delivery shows high efficacy with no side effects. An animal study of intranasally delivered CPM has shown that it does cross into the brain circulation. One study has assessed the systemic bioavailability and safety of a nasal spray solution developed to deliver doses of 1.12 and 2.24 mg CPM intranasally (0.4% nasal spray) and have found no adverse events [3].

Most notably, an article highlighted the CPM antiviral activity in various strains of influenza A/B, thus suggesting that it may have greater activity than the Food and Drug Administration (FDA) approved Tamiflu® [4]. 

Given the strong evidence supporting CPM’s antiviral and anti-inflammatory properties, we performed an in vitro study to test the virucidal effect of a nasal spray containing CPM as an active ingredient in addition to other excipients.

## Materials and methods

Ferrer Medical Innovations and Xlear Inc. developed and formulated the chlorpheniramine composition tested in this study (Table 1).

**Table 1:** Nasal spray composition Note: nasal spray composition with CPM at 0.4%: 3.6 mg/mL. Nasal spray composition has been developed by Ferrer Medical Innovations and formulated by Xlear Inc. CPM, chlorpheniramine maleate

Component
Purified water
CPM
Xylitol
Glycerin
Sodium bicarbonate

Procedure

Virus, Media, and Cells

The viral stock of SARS-CoV-2, USA-WA1/2020 strain, was prepared before testing through growth in Vero 76 cells. The culture medium for the prepared stock (test medium) was a minimum essential medium (MEM) with 2% fetal bovine serum and 50-µg/mL gentamicin.

Virucidal assay

The nasal spray was provided by the sponsor. As shown in Table 2, the compounds were mixed directly with the virus solution at a proportion of 90% compound preparation and 10% virus solution. A single concentration was tested in triplicate. The test medium without a virus was added to one tube of the prepared compound to serve as a toxicity control. Ethanol (70%) was tested in parallel as a positive control, and water only was tested as a virus control. The solution and virus were incubated at room temperature (22 ± 2°C) for 25 minutes. The solution was then neutralized through 1/10 dilution in the test medium.

Viral quantification

The surviving virus from each sample was quantified with standard end-point dilution assays. Briefly, samples were serially diluted 1/10 in the test medium. Then 100 µL of each dilution was plated into quadruplicate wells of 96-well plates containing 80-90% confluent Vero 76 cells. The plates were incubated at 37 ± 2°C under 5% carbon dioxide for six days. Each well was then scored for the presence or absence of the virus. The end-point titer 50% cell culture infection dose (CCID_50_) values were calculated using the Reed-Muench equation [5,6].

Statistical analysis

Three independent replicates of each sample were tested, and the average and standard deviation were calculated. Results were compared with untreated controls using one-way ANOVA (analysis of variance) with Dunnett’s multiple comparison test in GraphPad Prism (version 8) software.

Controls 

Virus controls were tested in water, and the reduction of the virus in the test wells compared with the virus control wells was calculated as the log reduction value (LRV). Toxicity controls were tested with a medium not containing the virus to determine whether the samples were toxic to cells. Neutralization controls were tested to ensure that viral inactivation did not continue after the specified contact time and that any residual sample in the titer assay plates did not inhibit the growth and detection of the surviving virus. We performed this procedure by adding toxicity samples to titer test plates and then spiking each well with a small amount of virus that would produce an observable cytopathic effect during the incubation period.

**Table 2:** Virucidal efficacy of nasal spray against SARS-CoV-2 after a 25-minute incubation with virus at 22 ± 2°C. ^a^Log10 CCID_50_ of virus per 0.1 mL, average of three replicates ± standard deviation ^b^LRV is the reduction of virus compared with that of the virus control. For wells with undetectable virus, a value equal to the lower limit of detection was assigned for statistical analyses. ***P < 0.001 by one-way ANOVA and Dunnett’s post-test compared with untreated virus control (water). LRV, log reduction value; CCID_50_, 50% cell culture infectious dose per mL; SARS-CoV-2, severe acute respiratory syndrome coronavirus 2; ANOVA, analysis of variance

	Concentration	Incubation	Virus Titer^a^	LRV^b^
Nasal spray	90%	25 minutes	1.7 ± 0.0	2.5***
Ethanol	67.5%	25 minutes	1.0 ± 0.6	3.2***
Virus control	NA	25 minutes	4.2 ± 0.4	NA

## Results

The results showing the viral titer and LRV values for SARS-CoV-2 after incubation with a single concentration of nasal spray are shown in Table 2. Toxicity was observed in the top dilution (1/10). The virus was observed below that dilution and therefore did not affect calculations of viral titer or LRV. After 25-minute contact time, the nasal spray reduced the levels of the virus from 4.2 to 1.7 log10 CCID_50_ per 0.1 mL, a statistically significant 2.5 LRV or equivalent of 99.7% reduction in COVID-19 virus titer. Neutralization controls demonstrated that the residual samples did not inhibit viral growth and detection in the endpoint titer assays. Virus controls and positive controls performed as expected.

## Discussion

In this study, we tested the virucidal activity of CPM using viral stock of SARS-CoV-2, USA-WA1/2020 strain in Vero 76 infected cells. The culture medium for the prepared stock (test medium) was MEM with 2% fetal bovine serum and 50-µg/mL gentamicin. The assay revealed that the nasal spray significantly reduced the levels of the virus. Hou et al. studied aspects of early infectivity and disease pathogenesis relevant to SARS-CoV-2 respiratory infections [7]. They found that the nose and proximal airways contained the highest percentage of angiotensin-converting enzyme 2 (ACE2) expressing ciliated cells and speculated that nasal surfaces might be the dominant initial site for SARS-CoV-2 respiratory tract infection [7]. Consequently, they suggested that complementary therapeutic strategies that reduce viral titer in the nose early in the disease may be beneficial [7]

CPM in an animal study shows potent inhibitory activity against divergent influenza A strains and one influenza B strain. It protects mice from the fatal challenge of the avian H7N9 influenza virus. It also attenuated the inflammatory responses and allergic syndromes caused by a viral infection [4]. In our in vitro study, a nasal spray formulation with CPM was able to reduce the equivalent of 99.7% of the COVID-19 virus titer. Intranasal delivery of CPM had fewer side effects than the enteral route [8,9]. Our study strengthens the antiviral and virucidal spectrum of CPM. The nasal spray containing CPM could become a cornerstone in the early treatment and prevention of viral infections, especially influenza A/B and COVID-19.

## Conclusions

This study demonstrates the strong virucidal effect against SARS-CoV-2 of a nasal spray containing CPM. Given the evidence supporting CPM’s antiviral and anti-inflammatory together with the results of this in vitro study, we propose to further study this formulation potential. Given that intranasally delivered CPM has broad antiviral effects with minimal side effects, our study could be extended to test other common respiratory viral infections.
